# High-Order Domain-Wall Dark Harmonic Pulses and Their Transition to H-Shaped and DSR Pulses in a Dumbbell-Shaped Fiber Laser at 1563 nm

**DOI:** 10.3390/mi16070727

**Published:** 2025-06-21

**Authors:** Alejandro Reyes-Mora, Manuel Durán-Sánchez, Edwin Addiel Espinosa-De-La-Cruz, Ulises Alcántara-Bautista, Adalid Ibarra-Garrido, Ivan Armas-Rivera, Luis Alberto Rodríguez-Morales, Miguel Bello-Jiménez, Baldemar Ibarra-Escamilla

**Affiliations:** 1 Instituto Nacional de Astrofísica, Óptica y Electrónica, Luis Enrique Erro, Sta. María Tonantzintla, Puebla 72824, Mexico; alejandro_rm@inaoep.mx (A.R.-M.); manueld@inaoep.mx (M.D.-S.); edwin_addiel@inaoep.mx (E.A.E.-D.-L.-C.); ulisesab@inaoep.mx (U.A.-B.); adalid.ibarra@inaoep.mx (A.I.-G.); ivan_rr1@hotmail.com (I.A.-R.); 2 Centro de Investigaciones en Óptica, A.C., Loma de Bosque 115, Col. Lomas del Campestre, León 37115, Mexico; beto7500@hotmail.com; 3 Instituto de Investigación en Comunicación Óptica, Universidad Autónoma de San Luis Potosí, Av. Karakorum, San Luis Potosí 78210, Mexico; miguel.bello@uaslp.mx

**Keywords:** high-order dark pulse, DSR pulse, h-shaped pulse, dual-wavelength, mode-locked fiber laser

## Abstract

In this work, we report the formation of multiple mode-locking states in an Erbium/Ytterbium co-doped fiber laser, such as domain-wall (DW) dark pulses, high-order dark harmonic pulses, dissipative soliton resonance (DSR) pulses, and dual-wavelength h-shaped pulses. By increasing the pump power and adjusting the quarter-wave retarder (QWR) plates, we experimentally achieve 310th-order harmonic dark pulses. DSR pulses emerge at a pump power of 1.01 W and remain stable up to 9.07 W, reaching a maximum pulse width of 676 ns and a pulse energy of 1.608 µJ, while Dual-wavelength h-shaped pulses have a threshold of 1.42 W and maintain stability up to 9.07 W. Using a monochromator, we confirm that these h-shaped pulses result from the superposition of a soliton-like pulse and a DSR-like pulse, emitting at different wavelengths but locked in time. The fundamental repetition rate for dark pulsing, DSR, and h-shaped pulses is 321.34 kHz. This study provides new insights into complex pulse dynamics in fiber lasers and demonstrates the versatile emission regimes achievable through precise pump and polarization control.

## 1. Introduction

Since their discovery, solitons generated in fiber lasers have attracted significant interest due to their wide-ranging applications in optical technologies [1,2]. Over the years, mode-locked (ML) fiber lasers have evolved considerably, with components such as the nonlinear optical loop mirror (NOLM), also known as the Sagnac interferometer, playing a pivotal role in their design. NOLMs offer ultrafast and highly stable switching capabilities, making them ideal for passive mode-locking (PML). A related component, the nonlinear amplifying loop mirror (NALM), incorporates rare-earth-doped fibers to enhance nonlinear-switching behavior. In particular, symmetric NOLM in conjunction with QWR plates has been shown to facilitate PML [3].

ML lasers are capable of emitting both bright and dark pulses, classified based on their energy distribution pattern. Bright pulses are characterized by a sharp intensity increase above the continuous wave (CW) background, while dark pulses manifest as abrupt intensity dips below the CW level. The coexistence of bright and dark pulses is primarily attributed to cross-phase modulation [4]. Both types of pulses are well described by the nonlinear Schrödinger equation (NLSE), while more complex behaviors, including dark vector solitons and Kerr-induced dark pulses, are captured by the complex Ginzburg–Landau equation (CGLE) [5,6,7,8,9,10].

The study of dark pulses has gained significant attention, as they exhibit reduced susceptibility to nonlinear dispersion effects, such as intrinsic Raman dispersion, and are less sensitive to ambient temperature fluctuations. These characteristics enable longer, and more stable pulse propagation compared to their bright counterparts. Dark pulse formation generally occurs via three primary mechanisms. The first involves the generation of NLSE-type dark pulses within a cavity operating under normal dispersion. These pulses typically exhibit a hyperbolic tangent intensity profile, one of the steady state solutions of the NLSE, and a single-peaked optical spectrum akin to bright solitons. Most lasers operating in this dark-pulse dynamic use Ytterbium-doped fibers as an active medium, hence, assuring operation in the normal dispersion regime.

The second mechanism introduces high-order nonlinearities into the laser cavity, for example, by adding a segment of highly nonlinear fiber (HNLF), which enhances the quintic nonlinearity. When these non-Kerr nonlinearities dominate, cubic-quintic NLSE (CQNLSE) dark pulses can emerge [11,12]. Depending on the cavity design, such pulses can arise in either anomalous or normal dispersion regimes.

The third mechanism is associated with DW-type dark pulses, which arise from the interaction of multiple wavelengths within the cavity. These interactions create temporal topological defects, leading to DW pulse generation in both dispersion regimes, as they are not limited by high-order nonlinearities. This process can result in polarization DW pulses [13,14,15] or double-wavelength DW pulses [16,17], which are typically characterized by multipeaked spectral profiles arising from the interplay of bright pulses at different wavelengths.

Broadly speaking, the formation of NLSE-type dark pulses is due to destructive wave interference in the normal dispersion regime. Such pulses typically present good stability and a narrow spectrum. As for the CQNLSE-type dark pulses, they are formed when the wave suffers an intensity drop due to the localized decrease in the refractive index with increasing intensity, while quintic nonlinearity stabilizes the pulses, avoiding collapse or dispersion. Their spectrum is wider than that of the NLSE type due to the quintic cubic interaction. Finally, the formation of DW-type dark pulses is due to an abrupt and localized transition between two different states with different phases and amplitudes. Their stability depends on the energy balance between the interacting states and is mainly provided by the pumping source, and the optical spectrum usually presents interference components. The temporal characteristics of the pulse are an intensity drop below a continuous wave background and are practically the same for the three cases.

Most works of DW dark pulse employ ring cavity configurations with anomalous dispersion, using the nonlinear polarization rotation (NPR) technique. In addition, various types of saturable absorbers have been explored for this purpose, including rare-earth-doped fiber sections, single-mode fiber (SMF), and graded-index multimode fiber (GIMF) arrays (e.g., the SMF-GIME-SMF configuration) [18,19,20], as well as 2D materials such as black phosphorus, carbon nanotubes, and molybdenum disulfide [21,22,23,24].

Table 1 summarizes several reports on DW dark pulse generation. These systems typically operate at low power with short cavity lengths (on the order of a few meters), yielding nanosecond-scale pulses with energies in the nanojoule range. Additionally, the generation of harmonic dark pulses is also of growing interest, with stable low-order harmonics and less stable, poorly defined high-order harmonics being reported [25,26,27].

In anomalous dispersion regimes, soliton formation is mainly governed by the balance between dispersion and the Kerr nonlinearity of the fiber. In contrast, under normal dispersion, solitons arise from the interplay among dispersion, Kerr nonlinearity, gain, loss, and gain dispersion. Dissipative soliton resonance (DSR) pulses formation occurs because of the balance between nonlinear gain saturation, losses, quintic nonlinearity, and spectral filtering due to the fiber’s birefringence. These pulses are distinctively rectangular in shape, with their width controllable via pump power adjustment while maintaining constant amplitude. Their optical spectra often feature sidebands, a characteristic signature of soliton dynamics.

The first experimental demonstrations of DSR pulses were reported in Erbium-doped fiber lasers (EDFLs), showing that DSR formation is independent of both the ML mechanism technique and the gain medium [28,29]. Later, the generation of DSR pulses in Ytterbium-doped fiber lasers was demonstrated, revealing the vectorial nature of the DSR pulses through careful tuning of the intra-cavity parameters [30,31]. While most DSR studies employ a ring cavity configuration, recent works have demonstrated that the DSR regime can also be achieved in alternative cavity designs—such as a Figure 8 laser—provided key parameters are properly adjusted [32,33].

DSR pulse dynamics—including harmonic generation, pulse motion and splitting, polarization effects, and multi-steady states—highlight the rich variety of operating regimes accessible by exploring the broad parameter space of DSR systems. This can be achieved by tuning the cavity dispersion, birefringence, polarization, and pump power [34,35,36,37,38,39,40,41]. Recently, fiber lasers operating in the DSR regime have also been used as pump sources for high-power laser systems [42,43,44,45].

Another interesting pulse regime is that of the dual-wavelength rectangular pulses, which can be generated in both normal and anomalous dispersion cavities, regardless of the gain medium. Dual-wavelength fiber lasers are of particular interest due to their applications in spectroscopy, fiber-optic sensing, and telecommunications. Erbium-doped fibers are commonly used as gain media in multi-wavelength laser systems, with tunable dual-wavelength operation often achieved through polarization-dependent loss control and the incorporation of Fabry–Perot filters combined with Bragg gratings [46,47,48,49,50].

The generation of h-shaped pulses can emit at one or two wavelengths, depending on the specific intracavity configuration. These pulses have garnered considerable attention in recent years, particularly in the ~1.5 µm spectral region. Their formation is strongly influenced by polarization state and pump power, often resulting in harmonic ML [51,52,53]. In normal dispersion cavities, h-shaped pulse emission has been shown to evolve in pulse bursts, indicating that the sharp leading edges of the h-shaped pulses are not solely governed by the peak power clamping (PPC) effect [54]. Under anomalous dispersion, increasing the cavity length strengthens harmonic generation [55,56].

Laser cavities incorporating Erbium (Er)- or Ytterbium (Yb)-doped fibers as gain media have shown transitions between single-pulse and h-shaped pulse emission, often facilitated by precise control over filtering mechanisms and the superposition of multiple filtering effects within the cavity [57,58,59]. Recently, wavelength-tunable h-shaped noise-like pulses were experimentally demonstrated [60]. Switching between various mode-locking regimes has been experimentally demonstrated for the first time in a dumbbell-shaped fiber laser through precise control of the polarization plates. DSR pulses, fundamental and high-order harmonic dark pulses, and h-shaped pulses have been achieved, and their evolution has been studied by increasing the pump power. This demonstrates that the cavity design is an ideal platform for studying complex pulses.

## 2. Experimental Setup

**Figure 1:**
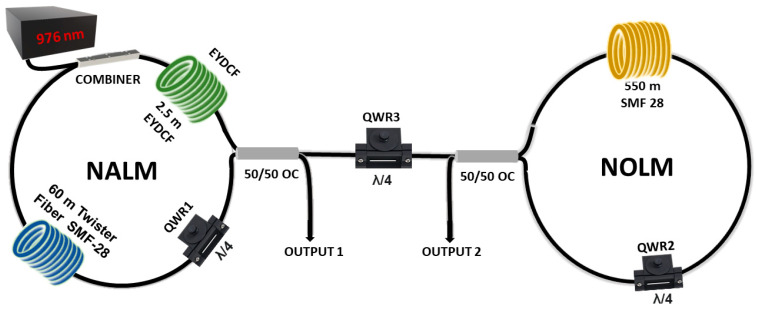
Diagram of the dumbbell-shaped fiber laser. EYDCF (Erbium-Ytterbium double-clad fiber), QWR (Quarter-Wave Retarder), OC (Optical coupler), SMF (Single mode fiber).

The experimental setup of the dumbbell-shaped Er/Yb-codoped fiber laser is shown in Figure 1. Fiber cavities designed to produce DW dark pulses typically include an interferometric mechanism, which, in the proposed configuration, is implemented by an NOLM.

The cavity consists of two main parts: a NOLM and a NALM. The NALM-based saturable absorber consists of 60 m of SMF-28 twisted fiber, twisted at a rate of 7 turns per meter. This twisting is applied to eliminate residual linear birefringence and to induce circular birefringence. The amplifier loop also features 2.5 m of EYDCF, which serves as the gain medium for pulse amplification. This fiber has a dispersion coefficient of −19 ps^2^/km and is pumped by a 976-nm laser source through a (2 + 1) × 1 pump combiner. A QWR1 plate is included in the NALM to manipulate the polarization state, thereby enhancing the NALM transmission. As for the NOLM, it is closed via a 50/50 optical coupler (OC) and includes a 550-m segment of SMF-28 twister fiber and a QWR1 plate.

The choice of a long fiber has critical implications for pulse formation due to the interaction of fiber nonlinearity and dispersion. A longer fiber allows for greater nonlinear phase accumulation, which is essential for the NOLM’s effective operation as a saturable absorber. However, it also contributes to significant pulse deformation if the pulse is too short. In addition, since nonlinear effects are enhanced, causing the appearance of new pulses and generating new dynamics. On the other hand, a NOLM with short fiber length does not offer sufficient nonlinearity to form pulses efficiently, leading to weak modulation.

The nonlinear mirrors are connected through one of their OC ports, thus forming a dumbbell-shaped cavity. A third QWR plate (QWR3) is placed in the linear cavity segment, whose purpose is to regulate the reflection between the two saturable absorber elements. The cavity design thus facilitates the tuning of the nonlinear transmission curve and allows the formation of several pulsing regimes, including fundamental and high-order harmonic dark pulses, DSR, and h-shaped pulses.

Total cavity length is approximately 644 m, and the net group delay dispersion is estimated to be −11.607 ps^2^, which indicates an average cavity dispersion of −20.72 ps^2^/km. Hence, the laser operates in a highly anomalous dispersive regime.

After verifying that output 1 and output 2 provide the same signal, both output ports were used to characterize the laser. From output 1, the temporal profile and optical spectrum were taken, while from output 2, the radio frequency spectrum was taken.

## 3. Results and Discussions

### 3.1. High-Order Harmonic Domain-Wall Dark Pulses

The generation of bright square pulses within the laser cavity can lead to the formation of dark square pulses through the DW phenomenon. For this mechanism to occur, the cavity must exhibit high nonlinearity to facilitate the coupling of the bright pulses. In our cavity setup, this condition is met using a long segment of SMF-28 fiber and applying high pump power. Initially, the generation of bright square pulses is achieved by adjusting the pump power between 1 and 9 W. Subsequently, by carefully tuning the QWR1 and QWR3 plates, the polarization state is altered to induce phase transitions that generate topological defects in the time domain—an essential condition for the emission of dark square pulses.

**Figure 2:**
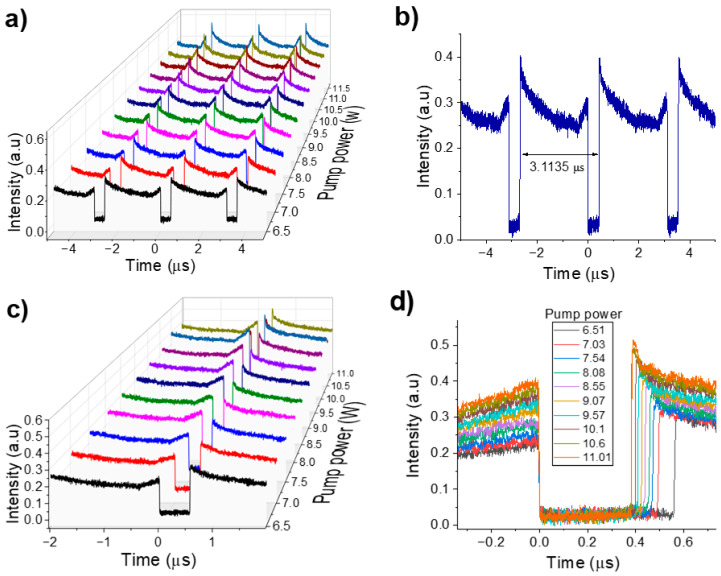
(**a**) Dark pulse train as a function of pump power, (**b**) single dark pulse train, (**c**) dark pulse evolution with respect to pump power, (**d**) pulse width reduction with increasing pump power from 6.51 to 11.01 W.

A similar amount of intensity in the counter-propagating beams within the NOLM coupler results in a higher contrast ratio in the transmission received by the NALM, as higher contrast favors the formation of dark square pulses. The dark pulse emission threshold occurs at a pump power of 6.5 W. As shown in Figure 2a, the dark pulse train remains stable up to a pump power of 11.01 W. Figure 2b shows that the period between adjacent pulses is 3.11 µs, corresponding to the cavity round-trip time. The repetition rate is 321.34 kHz, which is in accordance with the laser cavity length of 644 m.

An intensity peak observed on the trailing edge of the dark pulse, which decreases slightly before returning to the CW background, could be attributed to the relaxation oscillation in the gain medium when it experiences a sudden increase in intracavitary intensity.

Figure 2c,d show the reduction in pulse width as the pump power increases. Specifically, the pulse width decreases from 560 to 381 ns, experiencing a reduction of 179 ns when the pump power increases from 6.5 to 11.01 W. This behavior is linked to the increased saturation energy of the gain medium. As shown in Figure 2d, CW intensity rises with increasing pump power, primarily due to higher intra-cavity losses induced by the NOLM acting as a saturable absorber. Consequently, the CW power is temporally redistributed, enabling the cavity to absorb additional gain over successive round trips, which in turn leads to a higher intensity and a noticeable reduction in pulse duration.

The interplay of nonlinearity, dispersion, and birefringence—arising from the long SMF-28 fiber section and the QWR2 plate within the NOLM—introduces wavelength dependent intracavity loss. These losses lead to oscillations in the effective transmissivity of the cavity mirrors, leading to the formation of multiple wavelengths through nonlinear phase accumulation during the propagation of pulses. This process enables the coupling of significant pulse intensities, forming spectral peaks associated with bright pulses emitted at closely spaced wavelengths.

**Figure 3:**
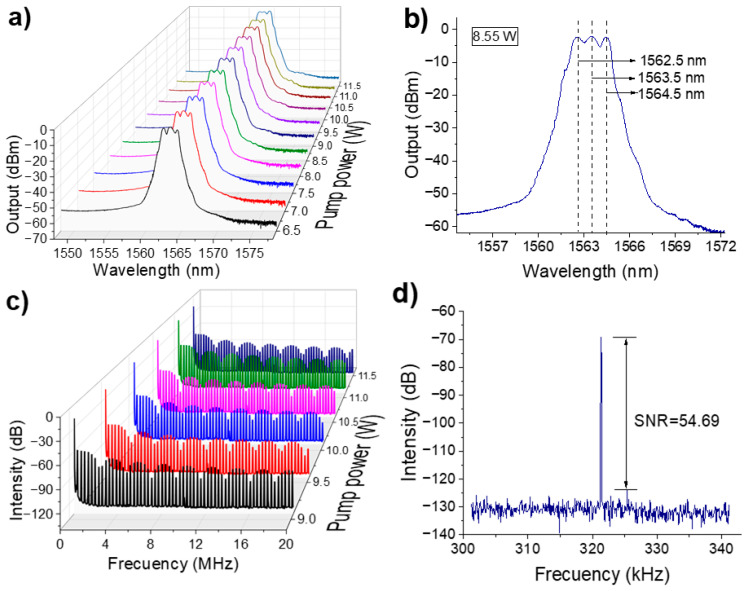
(**a**) Optical spectrum evolution when increasing pump power from 6.5 to 11.01 W, (**b**) Emission spectrum at a pump power of 8.55 W, (**c**) RF spectrum for different pump powers, (**d**) RF spectrum over a 40-kHz frequency span.

Figure 3a shows the evolution of the optical spectrum as a function of pump power. It can be seen that the spectral peaks begin to fade when increasing the pump power from 6.5 to 11.01 W, resulting in a smoother spectrum. This smoothing is primarily attributed to self-phase modulation within the cavity. As shown in Figure 3b, these spectral peaks are located equidistantly at 1 nm. At a pump power of 8.08 W, the 3-dB spectrum bandwidth is measured to be 3.75 nm. Figure 3c shows the radiofrequency (RF) spectrum over a wide range for different pump powers. As the pump power increases, the intensity modulation changes, showing an increasingly larger frequency period. This observation aligns well with the narrowing behavior previously shown in Figure 2d.

Although several wavelengths oscillate simultaneously within the cavity, only one frequency component is observed in the RF spectrum, as shown in Figure 3d. This confirms that the laser operates in a typical DW mode, where the topological defects induced by the three wavelengths traveling in the cavity generate only one frequency component. The RF peak is located at 321.34 kHz, which is consistent with the cavity round-trip length. Furthermore, the laser exhibits good stability, with a signal-to-noise ratio (SNR) of 54.69 dB.

Since the nonlinear phase shift is directly proportional to the intracavity light intensity, it plays a key role in enabling harmonic ML operation of dark pulses. This phase shift can be significantly enhanced either by increasing the pump power or by changing the polarization state within the cavity through the QWR1 and QWR2 plates.

Since the polarization plates induce birefringence, the polarization change that they produce in the Er/Yb dumbbell-shaped cavity is more pronounced, given the considerably long cavity length, unlike a shorter cavity, where this change has minimal effect on the formation of the harmonic pulses. In order to achieve high-order dark harmonic pulses, the pump power was set at 6.26 W, near the threshold of fundamental dark pulse generation. Besides, the QWR2 plate was positioned at the angle of greatest transmission of the NOLM, the QWR3 plate was set at the angle of maximum reflection between the two mirrors, and the QWR1 plate was set at a low transmission angle.

By increasing the transmission through the NALM and carefully adjusting the QWR1 plate, low-order dark harmonic pulses emerge. When the pump power is increased to 8.5 W, the NALM transmission is further enhanced, leading to the generation of higher-order dark harmonic pulses. This behavior is due to changes in intracavitary losses, which in turn modify the critical saturation power for ML, resulting in a shift to a new ML state.

**Figure 4:**
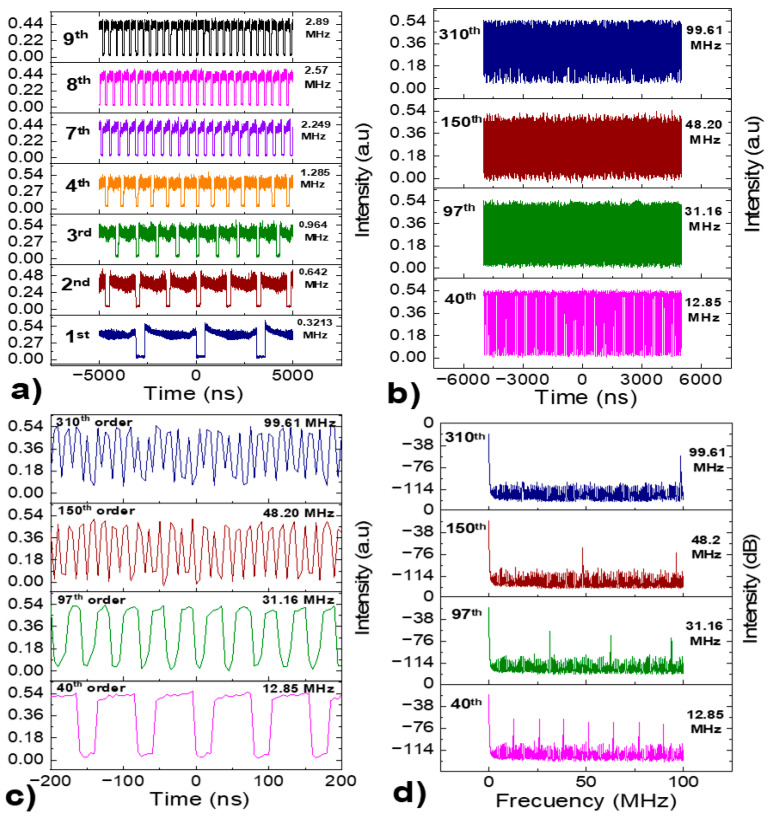
(**a**) Low-order dark harmonics, (**b**) High-order dark harmonics, with the 310th harmonic being the highest harmonic that can be stabilized, (**c**) close-up view of the pulses shown in (**b**–**d**) RF, spectrum corroborating the emission of different high-order dark harmonic pulse trains.

Starting from the fundamental emission state of the dark pulse, adjustments of the retarder plates and the pump power were able to shift the repetition frequency from 321.34 kHz to 99.61 MHz, while the pulse duration varied from 500 to 12 ns. Figure 4 shows different dark harmonic pulse trains that appear as the pump power and NALM transmission are varied.

In Figure 4a, the initial low-order dark harmonics are shown, exhibiting stable operation with rectangular pulse profiles that remain unaltered. Figure 4b displays the higher-order dark harmonic pulse trains, although individual pulses are not clearly distinguishable due to the wide temporal acquisition window.

Figure 4c shows a close-up view within a temporal window from −200 to 200 ns. The 40th and 97th dark harmonics maintain their square profile, although slight deformations are noticeable. As the harmonic order increases—from the 150th to the 310th—the rectangular shape of the dark pulses becomes progressively distorted, though pulse modulation remains discernible. At the 310th harmonic, the pulse shape can no longer abruptly fall or rise, occasionally emitting dark triangular pulses. This may be mainly due to detector saturation. The repetition frequency for each harmonic is measured using the RF spectrum analyzer, as shown in Figure 4d, to confirm that these emissions correspond to the reported dark harmonics.

### 3.2. DSR Pulses

When operating cavities with large net anomalous dispersion, it becomes easier to ensure that the DSR pulse is the dominant ML mechanism [61]. In our laser setup, DSR emission is achieved by adjusting the QWR1 and QWR2 plates to maximize transmission through both the NALM and NOLM, while the QWR3 plate is set to an angle that balances transmission and reflection between the two fiber loops. The laser threshold is obtained at a pump power of 1.42 W, and the DSR pulses are self-starting, provided wave plates remain fixed in their optimized positions.

The pulse train remains stable across different pump powers, with a pulse period of 3.11 µs, corresponding to the cavity round-trip time. DSR pulses are known to exhibit a square-shaped envelope with either a flat or sloped top [62]. In our case, the cavity filtering effect caused by the SMF-28 fiber’s birefringence influences the output pulse shape, contributing to the specific profile observed.

**Figure 5:**
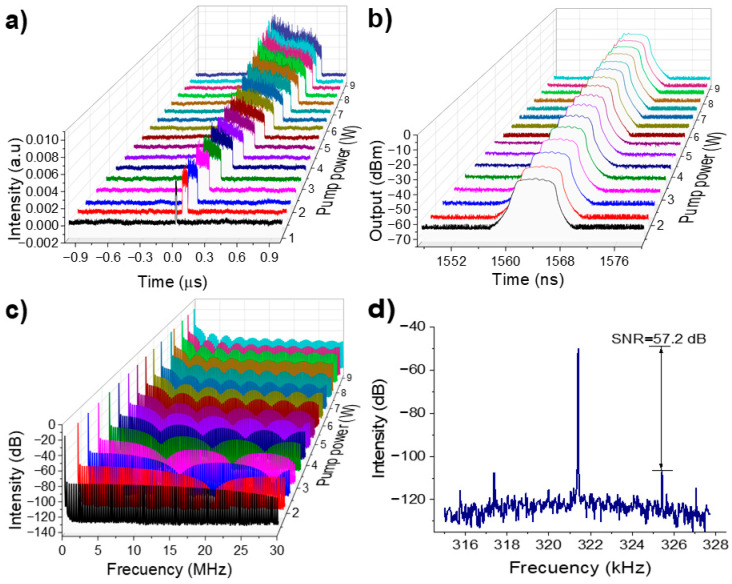
(**a**) Single pulse evolution as a function of the pump power, (**b**) Optical spectrum as a function of the pump power, (**c**) RF spectrum over a 30 MHz frequency range, (**d**) RF spectrum showing the fundamental beating note.

Figure 5a shows that the pulse width increases with increasing pump power, while the peak intensity remains almost constant, a behavior characteristic of the PPC effect. The pulse retains a nearly square profile with a slight downward edge at the tail. The pulse width increases monotonically from 11.92 to 676 ns as pump power rises from 1.42 to 9.07 W.

Figure 5b shows the evolution of the optical spectrum at different pump power levels. The central wavelength remains fixed at 1564 nm. A slight increase in 3-dB spectral width with pump power, exhibiting a flat-top spectral shape with multiple drifting peaks. This differs significantly from the smooth spectral profile typical of noise-like pulses or soliton rain/bunches spectra, which typically exhibit Kelly sidebands. Our DSR spectrum closely resembles that reported by K. Zhao et al. [63], where DSR was achieved in a highly anomalous dispersion regime (−14.08 ps^2^) under high pump powers, as in our case. In their case, autocorrelation traces confirmed the DSR nature of the pulses. These spectral small peaks may result from self-phase modulation, the interplay between dispersion and nonlinearity, or filtering effects induced within the laser cavity. The 3-dB spectral width does not remain constant but increases slightly with increasing pump power. The slight increase in the 3-dB spectral width, from 6.43 to 6.62 nm, is attributed to enhanced nonlinear phase accumulation with increasing pump power.

Figure 5c presents the spectral distribution over a 30 MHz frequency span with a 100 Hz resolution bandwidth. A well-defined envelope modulation is observed, with a frequency modulation period shortening as the pump power increases, confirming temporal pulse broadening by the Fourier transform property.

The RF spectrum in Figure 5d, measured over a frequency span of 13 kHz, shows a fundamental frequency at 321.34 kHz with a SNR of 57.2 dB, indicating excellent pulse train stability.

The DSR regime in our laser was stable over a wide range of pump powers, with no signs of pulse breaking. We did not observe soliton bunching, soliton rains, or any form of multi-soliton dynamics. This consistent behavior across various experimental conditions supports our identification of the pulses as DSR rather than chaotic soliton bunches or NLPR.

**Figure 6:**
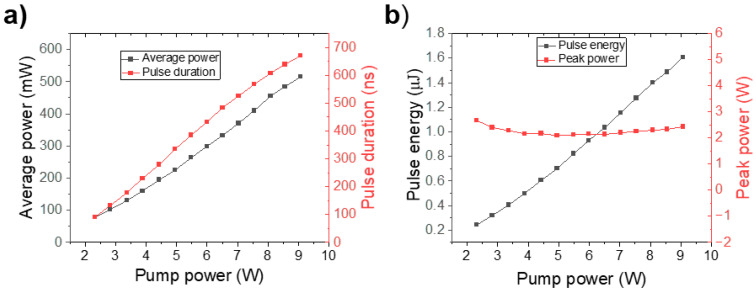
(**a**) Linear increase of pulse duration and estimated average power as functions of pump power in the same way as pulse energy, (**b**) while peak power remains almost constant.

According to DSR theory, as the pump power increases, the pulse width also increases, while the peak power remains nearly constant. This behavior is primarily attributed to reverse saturable absorption, where the transmittance decreases with increasing irradiance, effectively limiting the peak power. This phenomenon is illustrated in Figure 6a, which shows that both the pulse duration and the average output power increase linearly with increasing pump power. Figure 6b further confirms that the pulse intensity remains constant, indicating no change in peak power. In contrast, the pulse energy exhibits a linear increase with increasing pump power, demonstrating that square pulses with high pulse energy can be generated without the significantly high peak power typical of DSR pulses.

### 3.3. Dual Wavelength H-Shaped Pulses

Most fiber lasers that emit dual-wavelength h-shaped pulses typically use Thulium-doped fibers; however, there have also been reports of dual-wavelength emission in Er- or Er/Yb-doped fibers, in both normal and anomalous dispersion laser cavities. Bidirectional structures, such as the one used in our experimental research, combine two pulses traveling in opposite directions. This results in a notable difference between the central wavelengths of the counter-propagating pulses, influenced by cavity dispersion.

The generation of the dual-wavelength h-shaped pulse is likely due to the cavity spectral filtering effect provided by the NALM’s birefringence and to the asymmetric gain shaping. Birefringence induced by specific adjustments of the QWR1 plate causes the filter to favor a second wavelength (1542.8 nm), which can be amplified due to the broad gain bandwidth of the EYDF. The two pulses, at 1542.8 nm and 1559.5 nm, travel as a unit presumably due to nonlinear coupling due to the effects of SPM and XPM. Nonlinear interactions such as SPM and XPM introduce modulations or interferences that shape the temporal part of the pulse. A discussion on the formation of dual-wavelength h-shaped pulses by changing the filtering effects and NPR is presented by Gu et al. [59]. References [57,58] complement this information.

The formation of dual-wavelength h-shaped pulses occurs when the QWR2 plate of the NOLM is set at the angle of maximum transmission toward the NALM, while the QWR1 plate of the NALM is adjusted to increase transmission toward the NOLM. Additionally, the QWR3 plate is positioned at a balanced point between the transmission and reflection within the nonlinear mirrors. This dual-wavelength emission is achieved starting at a pump power of 1.42 W and remains stable up to 9.07 W. By correctly positioning the retarder plates, the corresponding adjustment in the cavity birefringence causes rotation of the intracavity polarization state, which helps balance the gain and losses to achieve dual-wavelength lasing.

Due to the long length of the NOLM, a large nonlinear phase shift is generated even at low powers, contributing to the PPC effect. This, combined with weak birefringence, promotes the formation of the h-shaped pulse, exhibiting similarities with DSR pulses. Theoretically, rotating the retarder plates modifies local intracavity birefringence, affecting pulse characteristics.

**Figure 7:**
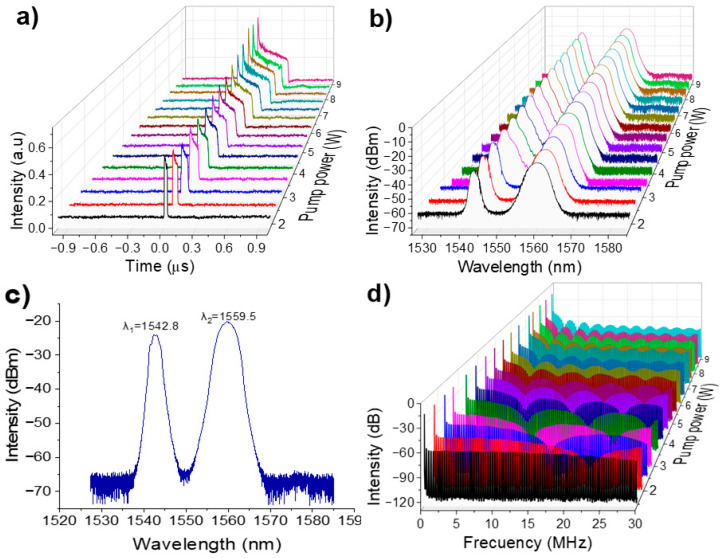
(**a**) h-shaped pulse evolution as a function of pump power, (**b**) dual-wavelength spectrum emission with respect to pump power, (**c**) single optical spectrum profile at 6.5 W, (**d**) RF spectrum over a 30 MHz frequency range.

Figure 7a illustrates the h-shaped pulse profile at various pump power. As the pump power increases, the h-shaped pulse exhibits an increase in pulse duration. Since there is no saturation in gain of the cavity, the pulse width continues to increase steadily.

Figure 7b demonstrates the dual-wavelength nature of the h-shaped pulsed emission. As the pump power rises, the optical spectrum remains unchanged, maintaining its dual-wavelength output. The short wavelength in the optical spectrum features a soliton profile, while the long wavelength corresponds to a DSR pulse profile. Figure 7c shows the dual-wavelength optical spectrum at a pump power of 6.5 W, with the short-wavelength emission centered at λ_1_ = 1542.8 nm and a 3-dB bandwidth of 3.26 nm, and the long-wavelength component centered at λ_2_ = 1559.5 nm with a 3-dB bandwidth of 6.92 nm.

Figure 7d illustrates the RF spectrum over a 30-MHz frequency span, showing modulation similar to that observed for DSR pulses, though not fully refined due to the pronounced intensity peak at the leading edge of the h-shaped pulse. The high stability of this fundamental ML regime is confirmed by the measured SNR of 57.03 dB at 321.34 kHz.

**Figure 8:**
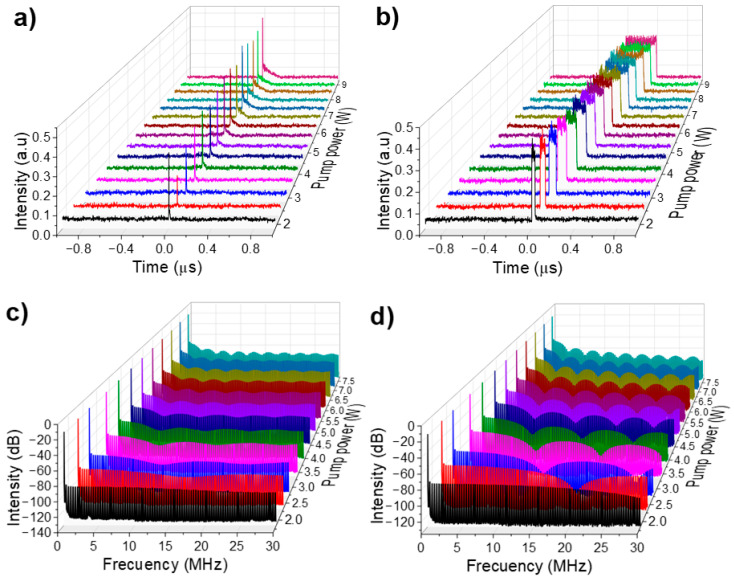
(**a**) Temporal emission at λ_1_ = 1542.8 nm filtered with the monochromator, (**b**) Temporal emission at λ_2_ = 1559.5 nm filtered with the monochromator, (**c**) RF spectrum for the λ_1_ = 1542.8 nm emission filtered with the monochromator, (**d**) RF spectrum for the λ_2_ = 1559.5 nm emission filtered with the monochromator.

The emission corresponding to each of the two wavelengths was isolated to further analyze the pulse structure using a monochromator to observe their respective pulse profiles. Figure 8a shows the pulse profile for λ_1_ = 1542.8 nm. As the pump power rises, the pulse intensity rises significantly, while its base broadens in time, causing the pulse profile to acquire an almost triangular shape at its base. This effect is likely due to the bandwidth limit of the monochromator filter, which does not fully suppress the DSR spectral component, giving the pulse this appearance. Referring to the complete h-shaped pulse, this corresponds to the intense, sharp peak’s leading edge. In this case, the PPC effect contributes minimally to the formation of the DSR pulse but helps prevent pulse splitting by broadening it.

In contrast, the pulse profile at λ_2_ = 1559.5 nm, shown in Figure 8b, behaves like a DSR pulse. It shows an increase in pulse duration with nearly constant intensity. Figure 8c presents the RF spectrum for the emission at λ_1_ = 1542.8 nm. As the pump power increases, the frequency modulation becomes more defined, reflecting the broadening of the pulse base and its evolving shape. Figure 8d shows the measured RF spectrum for the emission at λ_2_ = 1559.5 nm. In this case, the frequency modulation is well-defined, clearly indicating the broadening of the DSR square pulse. The SNR was measured for both wavelengths, demonstrating excellent stability and confirming the same fundamental repetition rate of 321.34 kHz.

The combination of gain depletion—which induces asymmetric deformation during pulse formation—and the modulation introduced by NOLM contributes to the h-shaped profile of the pulses. Based on the experimental results, it was confirmed that the dual-wavelength h-shaped pulses are generated through the combined filtering effects of the NOLM, NALM, and the NPR within the cavity. This results in two overlapping pulses that exhibit no temporal separation on the oscilloscope [59].

**Figure 9:**
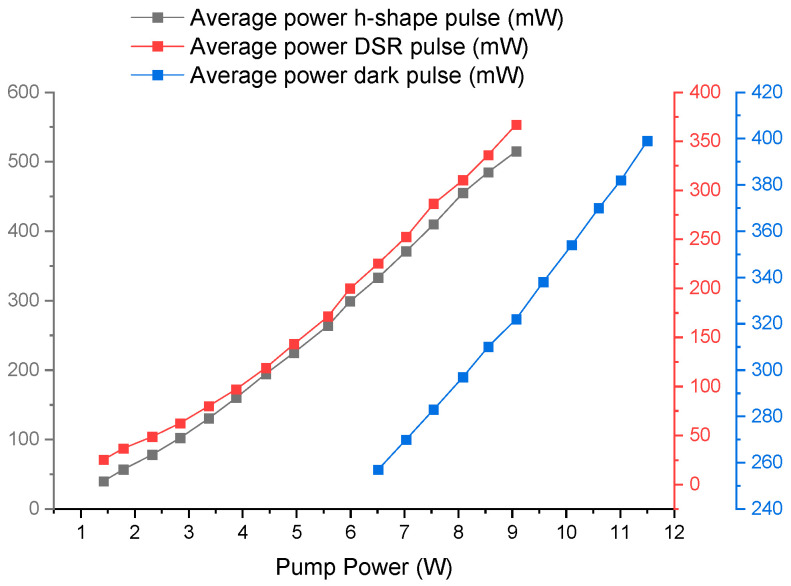
The average powers for the three reported dynamics are graphed, showing linear behavior with pumping power for each of them.

It is important to note the average power for the three dynamics studied for a possible future study or for some application. Figure 9 shows how the average power increases linearly with the pump power. It is notable that the average power of the dark pulses is moderately low compared to the other two dynamics since the energy is distributed over a continuous wave background.

Although pumping efficiencies in fiber lasers can be high, in our case, the efficiency is low due to the long length of the laser cavity, where much of the pump energy is lost, generating unwanted spontaneous light. Similarly, unwanted nonlinear effects, thermal issues, and coupling losses drain useful energy from the laser and lower its overall efficiency.

## 4. Conclusions

To the best of our knowledge, this is the first report of dark square pulses and high-order dark harmonics observed in a dumbbell-shaped fiber laser, achieving stable emission up to the 310th harmonic order. Unlike previously reported cavities for dark harmonic pulse emission, our cavity was built without the need for sophisticated saturable absorbers or specialized fibers such as HNLF. The NOLM functions as a saturable absorber to enable mode locking, and, due to the asymmetric amplification NALM structure within the cavity, it allows the generation of high repetition rates. The QWR1 plate, positioned in the NALM, controls the transmission to the NOLM, allowing the generation of various pulsed regimes, such as fundamental dark pulses, high-order harmonic dark pulses, DSR pulses, and h-shaped pulses.

The dark pulses and their harmonics remained stable with pump powers ranging from 6.5 to 11.01 W, during which the repetition rate increased from the fundamental repetition rate of 321.34 kHz to the 310th-order harmonic at 99.61 MHz. RF spectrum measurements were performed to confirm proper harmonic emission. DSR emission was achieved at pump powers between 1.01 and 9.07 W. For these dynamics, transmission in the NALM must be maximized, while transmission in the NOLM remains low.

The emission wavelength for both bright and dark pulses was ~1564 nm. The generation of h-shaped pulses takes place when the transmission in the NOLM is high and the transmission in the NALM is slightly more than half its maximum. It was found that h-shaped pulses result from the coexistence of two pulses at different wavelengths: one exhibiting a soliton profile and the other a DSR profile.

The proposed multi-functional laser source’s applicability is broad, since due to advantages like high stability through propagation, and rectangular pulse shaping, the rectangular dark pulses obtained in this laser configuration could have potential applications in fields like advanced optical communications, optical signal processing, and sensing systems; and the dual-wavelength h-shaped pulse operational regime, could be implemented in fields like spectroscopy.

## Figures and Tables

**Table 1:** Summary of main experimental works regarding DW dark pulse generation in ML fiber lasers.

Cavity Config.	Saturable Absorber	Pump Power (mW)	Repetition Rate (MHz)	Pulse Width (ns)	Output Power (mW)	Pulse Energy (nJ)	Emission Wavelength (nm)	Year	Ref.
Ring	NPR	260	18.18	4.5	40	2.2	1588–1591	2011	[16]
Ring	Graphene oxide	200	0.41	90.8	0.858	-	1062.5	2014	[6]
Ring	MoS_2_	242	17.4	-	0.80	0.046	1037, 1039	2016	[23]
Ring	NPR	1100	5.854	11.8	-	-	1880, 1900	2017	[17]
Ring	Black P.	90	14.6	16.95	8.61	-	1596, 1599	2018	[21]
Ring	Bi_2_Te_3_	80	20.7	10.3	-	-	1956, 1958	2019	[22]
Ring	Glycerin	33.5	23.08	-	33.5	-	1569.4	2019	[7]
Ring	TDF	120	3.03	18	-	-	1566.75	2019	[19]
Ring	TDF	120	2.96	100	4.14	1.4	1566.34	2019	[25]
Figure 9	NALM	225	0.323	11.8	8.8	27.2	1556, 1563	2019	[27]
Linear	NOLM	362	0.203	336	0.10	49	1041.5	2022	[13]
Ring	MWT	89	5.79	25	25	-	1562, 1563 1564	2023	[8]
Ring	TCTDF	112	0.96	250	6.58	3.24	1556, 1557, 1558	2024	[26]
Ring	SMF-GIME-SMF	97	21.5	5	16.6	769	1567.2 1569.4	2024	[18]
dumbbell	NOLM-NALM	6500	0.3213	560	112	348	1562.5 1563.5 1564.5	2025	This work

## Data Availability

The original contributions presented in this study are included in the article. Further inquiries can be directed to the corresponding author.
